# Target regimen profiles for tuberculosis treatment

**DOI:** 10.2471/BLT.24.291881

**Published:** 2024-05-28

**Authors:** Christian Lienhardt, Kelly E Dooley, Payam Nahid, Charles Wells, Theresa S Ryckman, Emily A Kendall, Gerry Davies, Grania Brigden, Gavin Churchyard, Daniela Maria Cirillo, Eugenia Di Meco, Ramya Gopinath, Carole Mitnick, Cherise Scott, Farhana Amanullah, Cathy Bansbach, Martin Boeree, Michael Campbell, Francesca Conradie, Angela Crook, Charles L Daley, Keertan Dheda, Andreas Diacon, Agnes Gebhard, Debra Hanna, Norbert Heinrich, Anneke Hesseling, David Holtzman, Mathilde Jachym, Peter Kim, Christoph Lange, Lindsay McKenna, Graeme Meintjes, Norbert Ndjeka, Nguyen Viet Nhung, Bern-Thomas Nyang’wa, Nicholas I Paton, Raghuram Rao, Michael Rich, Rada Savic, Ingrid Schoeman, Boitumelo Semete Makokotlela, Mel Spigelman, Eugene Sun, Elin Svensson, Phumeza Tisile, Francis Varaine, Andrew Vernon, Mukadi Ya Diul, Tereza Kasaeva, Matteo Zignol, Medea Gegia, Fuad Mirzayev, Samuel G Schumacher

**Affiliations:** a French National Research Institute for Sustainable Development, Montpellier, France.; b Division of Infectious Diseases, Vanderbilt University Medical Center, Nashville, United States of America (USA).; c Institute for Global Health, University of California San Francisco, San Francisco, USA.; d Bill & Melinda Gates Medical Research Institute, Boston, USA.; e Infectious Disease Division, School of Medicine, John Hopkins University, Baltimore, USA.; f Institute of Infection and Global Health, University of Liverpool, Liverpool, England.; g The Global Fund to Fight AIDS, Tuberculosis and Malaria, Geneva, Switzerland.; h Aurum Institute, Johannesburg, South Africa.; i Istituto di Ricovero e Cura a Carattere Scientifico (IRCCS), San Raffaele Scientific Institute, Milan, Italy.; j European Medicines Agency, Amsterdam, Kingdom of the Netherlands.; k Division of Anti-Infectives, Food and Drug Administration, Washington DC, USA.; l School of Medicine, Harvard Medical School, Boston, USA.; m Unitaid, Geneva, Switzerland.; n Indus Hospital, Karachi, Pakistan.; o ChinaCat Enterprises, Gig Harbor, USA.; p University Medical Center, Radboud University, Amsterdam, Kingdom of the Netherlands.; q Clinton Health Access Initiative, Boston, USA.; r Wits Health Consortium, Johannesburg, South Africa.; s MRC Clinical Trials Unit, University College of London, London, England.; t Division of Mycobacterial and Respiratory Infections, National Jewish Health, Denver, USA.; u Centre for Lung Infection and Immunity, University of Cape Town, Cape Town, South Africa.; v TASK, Cape Town, South Africa.; w KNCV, The Hague, Kingdom of the Netherlands.; x Global Health Division, Bill & Melinda Gates Foundation, Seattle, USA.; y Division of Infectious Diseases and Tropical Medicine, LMU University Hospital, LMU, Munich, Germany.; z Desmond Tutu TB Centre, Stellenbosch University, Stellenbosch, South Africa.; aa Centre Hospitalier de Bligny, Fontenay-lès-Briis, Paris, France.; ab Division of AIDS, National Institute of Allergy and Infectious Diseases, Bethesda, USA.; ac Research Center Borstel, Borstel, Germany.; ad Treatment Action Group, New York, USA.; ae Department of Medicine, University of Cape Town, Cape Town, South Africa.; af National Department of Health, Pretoria, South Africa.; ag Vietnam Medical Association, Hanoi, Viet Nam.; ah Médecins Sans Frontières, Geneva, Switzerland.; ai Yong Loo Lin School of Medicine, National University of Singapore, Singapore.; aj Indian Ministry of Health and Family Welfare, New Delhi, India.; ak Partners In Health, Boston, USA.; al TB Proof, Cape Town, South Africa.; am South African Health Products Regulatory Authority, Pretoria, South Africa.; an TB Alliance, New York, USA.; ao Médecins Sans Frontières, Paris, France.; ap National Institute of Allergy and Infectious Diseases, Bethesda, USA.; aq United States Agency for International Development, Washington DC, USA.; ar Global Tuberculosis Programme, World Health Organization, Avenue Appia 20, 1202 Geneva, Switzerland.

## Abstract

Simpler, shorter, safer and more effective treatments for tuberculosis that are easily accessible to all people with tuberculosis are desperately needed. In 2016, the World Health Organization (WHO) developed target regimen profiles for the treatment of tuberculosis to make drug developers aware of both the important features of treatment regimens, and patient and programmatic needs at the country level. In view of recent ground-breaking advances in tuberculosis treatment, WHO has revised and updated these regimen profiles. We used a similar process as for the 2016 profiles, including a baseline treatment landscape analysis, an initial stakeholder survey, modelling studies estimating the impact and cost-effectiveness of novel tuberculosis treatment regimens, and an extensive stakeholder consultation. We developed target regimen profiles for the treatment of rifampicin-susceptible and rifampicin-resistant tuberculosis, as well as a pan-tuberculosis regimen that would be appropriate for patients with any type of tuberculosis. We describe the revised target regimen profile characteristics, with specific minimal and optimal targets to be met, rationale and justification, and aspects relevant to all target regimen profiles (drug susceptibility testing, adherence and forgiveness, treatment strategies, post-tuberculosis lung disease, and cost and access considerations). We discuss the trade-offs of proposed characteristics for decision-making at developmental or operational levels. We expect that, following these target regimen profile revisions, tuberculosis treatment developers will produce regimens that are quality-assured, affordable and widely available, and that meet the needs of affected populations.

## Résumé

Des traitements de la tuberculose plus simples, plus courts, plus sûrs et plus efficaces, facilement accessibles à toutes les personnes atteintes de tuberculose, font cruellement défaut. En 2016, l’Organisation mondiale de la santé (OMS) a élaboré des profils de schéma thérapeutique cible pour le traitement de la tuberculose, afin de sensibiliser les concepteurs de médicaments aux caractéristiques importantes des schémas thérapeutiques et aux besoins des patients et des programmes au niveau national. Compte tenu des avancées récentes dans le traitement de la tuberculose, l’OMS a révisé et mis à jour ces profils de schéma thérapeutique. Nous avons appliqué un processus similaire à celui des profils de 2016, y compris une analyse de base des différentes possibilités thérapeutiques, une enquête initiale auprès des parties prenantes, des études de modélisation estimant l’impact et le rapport coût-efficacité des nouveaux schémas thérapeutiques pour la tuberculose, ainsi qu’une vaste consultation des parties prenantes. Nous avons élaboré des profils de schéma thérapeutique cible pour le traitement de la tuberculose sensible à la rifampicine ou résistant à la rifampicine, ainsi qu’un schéma multiforme qui conviendrait aux patients atteints de n’importe quel type de tuberculose. Nous décrivons les caractéristiques du profil révisé de schéma thérapeutique cible, avec les objectifs minimaux et optimaux spécifiques à atteindre, le raisonnement et les aspects pertinents pour tous les profils de schéma thérapeutique cible (tests de sensibilité aux médicaments, observance thérapeutique et manque d’observance («forgiveness»), stratégies de traitement, maladie pulmonaire post-tuberculeuse et considérations de coût et d’accès). Nous discutons des compromis des caractéristiques proposées pour la prise de décisions au niveau du développement ou au niveau opérationnel. Nous espérons qu’à la suite de ces révisions du profil de schéma thérapeutique cible, les concepteurs de traitements antituberculeux produiront des schémas dont la qualité est assurée, qui sont abordables et largement disponibles et qui répondent aux besoins des populations touchées.

## Resumen

Se necesitan con urgencia tratamientos más sencillos, breves, seguros y eficaces contra la tuberculosis que sean fácilmente accesibles para todas las personas con tuberculosis. En 2016, la Organización Mundial de la Salud (OMS) elaboró perfiles objetivo de esquemas terapéuticos para el tratamiento de la tuberculosis con el fin de que los fabricantes de medicamentos conocieran tanto las características importantes de estos esquemas como las necesidades programáticas y de los pacientes en cada país. Teniendo en cuenta los recientes avances pioneros en el tratamiento de la tuberculosis, la OMS ha revisado y actualizado estos perfiles de esquemas terapéuticos. Se ha seguido un proceso similar al de los perfiles de 2016, que incluye un análisis de referencia del panorama terapéutico, una encuesta inicial a las partes interesadas, estudios de modelización para estimar el impacto y la rentabilidad de los nuevos esquemas terapéuticos para el tratamiento de la tuberculosis, y una amplia consulta a las partes interesadas. Se desarrollaron perfiles objetivo de esquemas terapéuticos para el tratamiento de la tuberculosis sensibles a la rifampicina y resistente a la rifampicina, así como un esquema farmacológico capaz de tratar todas las formas de tuberculosis que sería apropiado para pacientes con cualquier tipo de tuberculosis. Se describieron las características revisadas de los perfiles objetivo de los esquemas terapéuticos, con los objetivos mínimos y óptimos específicos que deben alcanzarse, los fundamentos y la justificación, y los aspectos relevantes para todos los perfiles objetivo de los esquemas terapéuticos (pruebas de sensibilidad a los fármacos, adherencia y olvido, estrategias de tratamiento, enfermedad pulmonar postuberculosa, y consideraciones de coste y acceso). Se discutieron las ventajas y desventajas de las características propuestas para la toma de decisiones a nivel de desarrollo u operativo. Se espera que, tras estas revisiones de los perfiles objetivo de los esquemas terapéuticos, las personas encargadas del desarrollo de tratamientos para la tuberculosis elaboren esquemas terapéuticos de calidad garantizada, asequibles y ampliamente disponibles, y que respondan a las necesidades de las poblaciones afectadas.

## ملخص

هناك حاجة ماسة إلى علاجات أكثر بساطة، وأقصر مدة، وأكثر أمانًا وفعالية لمرض السل، والتي يمكن لجميع الأشخاص المصابين بالسل الحصول عليها بسهولة. قامت منظمة الصحة العالمية (WHO) في عام 2016 بتطوير جوانب النظام المستهدف لعلاج السل، بهدف توعية مطوري الأدوية بالخصائص المهمة لأنظمة العلاج، واحتياجات المرضى والبرامج على مستوى الدولة. وفي ضوء التطورات الهائلة الأخيرة في علاج السل، قامت منظمة الصحة العالمية بمراجعة جوانب هذا النظام وتحديثها. قمنا باتباع عملية مشابهة لتلك المستخدمة في جوانب عام 2016، بما في ذلك تحليل الواقع الأساسي للعلاج، ومسح أولي لأصحاب المصلحة، ودراسات وضع النماذج التي تضع تقديرات لتأثير نظم علاج السل الجديدة وفعاليتها من حيث التكلفة، واستشارات مكثفة مع أصحاب المصلحة. كما قمنا بتطوير جوانب نظام العلاج المستهدف لحالات السل الحساس للريفامبيسين والسل المقاوم للريفامبيسين، بالإضافة إلى نظام علاج السل الشامل الذي قد يكون مناسبًا للمرضى بأي نوع من أنواع السل. نحن نصف الخصائص المنقحة لجوانب النظام المستهدف، مع أهداف الحد الأدنى المثلى المحددة التي يجب تحقيقها، والأساس المنطقي والمبرر، والنقاط المتعلقة بكل جوانب النظام المستهدف (اختبار الحساسية للأدوية، والالتزام والتهاون، واستراتيجيات العلاج، وأمراض الرئة ما بعد السل، و اعتبارات التكلفة والحصول عليها). نناقش الموازنة بين الخصائص المقترحة لصنع القرار على مستويات التطوير أو التشغيل. ونتوقع أنه بعد هذه المراجعات لجوانب النظام المستهدف، فإن مطوري علاج السل سيقومون بوضع أنظمة مضمونة الجودة، وبأسعار معقولة، ومتاحة على نطاق واسع، وتلبي احتياجات السكان المعنيين.

## 摘要

迫切需要更简单、疗程更短、更安全、更有效且所有结核病患者都能轻易获得的结核病治疗方法。2016 年,世界卫生组织 (WHO) 制定了结核病治疗的目标方案概况,以便药物开发人员了解治疗方案的重要特征以及国家层面的患者和规划需求。鉴于最近在结核病治疗方面取得的突破性进展,世卫组织对这些治疗方案概况进行了修订和更新。我们使用了与 2016 年概况类似的流程,包括基线治疗前景分析、对利益相关者的初步调查、估计新型结核病治疗方案的影响和成本效益的建模研究,以及咨询广泛的利益相关者。我们制定了治疗利福平敏感和利福平耐药结核病的目标方案概况,以及适用于任何类型结核病患者的泛结核病治疗方案。我们介绍了修订后的目标治疗方案概况特征,包括要达到的最低和最佳具体目标、基本原理和理由,以及与所有目标治疗方案概况相关的方面(药敏试验、依从性和宽恕心理、治疗策略、结核后肺疾病,以及成本和可获得性考虑因素)。我们讨论了在药物开发或运营层面进行决策时对拟议特征所做权衡。我们期望,在这些目标治疗方案概况修订之后,结核病治疗开发人员能够制定出质量可靠、价格合理且广泛可用的治疗方案,从而满足受影响人群的需求。

## Резюме

Крайне необходимы более простые, короткие, безопасные и эффективные методы лечения туберкулеза, которые были бы легко доступны для всех больных туберкулезом. В 2016 году Всемирная организация здравоохранения (ВОЗ) разработала целевые профили схем лечения туберкулеза, чтобы разработчики лекарств знали как о важных особенностях схем лечения, так и о потребностях пациентов и программ на уровне стран. С учетом последних новаторских достижений в области лечения туберкулеза ВОЗ пересмотрела и обновила эти профили схем. Использовался тот же процесс, что и при составлении профилей 2016 года, включая анализ базовой ситуации с лечением, первоначальный опрос заинтересованных сторон, модельные исследования, оценивающие влияние и экономическую эффективность новых схем лечения туберкулеза, и широкие консультации с заинтересованными сторонами. Разработаны целевые профили схем лечения туберкулеза, чувствительного к рифампицину и устойчивого к рифампицину, а также пантуберкулезная схема, которая подходит для пациентов с любым типом туберкулеза. В статье приводится описание пересмотренных характеристик целевых схем с указанием конкретных минимальных и оптимальных целей, которые должны быть достигнуты, их обоснование и подтверждение, а также описываются аспекты, относящиеся ко всем целевым схемам (тестирование на восприимчивость к препаратам, приверженность лечению и устойчивость результатов в случае нарушения режима, стратегии лечения, посттуберкулезные заболевания легких, а также принятие во внимание стоимости и доступа). Также обсуждаются компромиссы между предлагаемыми характеристиками для принятия решений на уровне разработки или оперативного управления. Ожидается, что после пересмотра целевых профилей схем разработчики препаратов для лечения туберкулеза будут разрабатывать такие схемы, которые будут гарантированно качественными, доступными и широко распространенными и будут отвечать потребностям затронутых групп населения.

## Introduction

Significant progress has been achieved over the last two decades in the development of tuberculosis treatment, with the discovery of new chemical entities and trials testing new combinations of drugs. This progress has resulted in substantial improvements in the treatment of tuberculosis, and the World Health Organization (WHO) has recommended shortened treatment regimens for drug-susceptible and drug-resistant tuberculosis.^1^^–^^3^ With new chemical entities currently transitioning into clinical testing, the possibility of more efficacious, shorter and safer regimens for all forms of tuberculosis now appears to be achievable.^4^

The early 2000s brought the discovery of new chemical entities with new modes of action on tuberculosis bacilli,^5^^,^^6^ such as the development of diarylquinolines and nitroimidazoles, and the repurposing of active compounds traditionally used against Gram-positive infections (oxazolidinones);^7^ all these developments played a major role in advancing new treatment options for drug-resistant tuberculosis. In parallel, WHO proposed new approaches to shorten the lengthy development pathway, breaking away from the classical approach of first testing single new compounds and then identifying the most suitable drug combination.^8^^,^^9^

In 2017, WHO published *Target regimen profiles for treatment of tuberculosis*, promoting an end-to-end process integrating the development of new drugs within the development of new regimens, and defining specific requirements that new regimens should meet.^10^ In view of the ground-breaking progress in tuberculosis treatment over recent years, and the subsequent transformations in the recommended standards of care by WHO, it is necessary to revise and update these target regimen profiles. 

## Methods

The revision of the target regimen profiles for tuberculosis treatment by WHO followed similar methods to those established for initial regimen development in 2016. The WHO Global Tuberculosis Programme established a Scientific Target Regimen Profile Development Group that included leading scientists and experts, public health officials, regulators, donors, programme managers and representatives of civil society organizations. Our group served to support the entire development process by reviewing drafts at several stages, contributing to discussions during meetings and assisting with the writing process.

After a critical review of the 2016 document, our group retained the initial approach of classifying target regimen profiles for rifampicin-susceptible and rifampicin-resistant tuberculosis. Further, considering the possibility of developing a regimen composed of entirely new anti-tuberculosis drugs for which minimal or no resistance would exist, we maintained the pan-tuberculosis profile. We conducted a stakeholder survey during May–July 2022 to assess the use of the 2016 target regimen profiles, evaluate the relevance of the proposed regimen characteristics, prioritize these characteristics and evaluate potential trade-offs. The WHO Global Tuberculosis Programme commissioned two complementary modelling studies to inform our discussion. The first study estimated the potential health impact of novel treatments on the outcome of cure, considering various regimen characteristics such as treatment efficacy, duration, ease of adherence and forgiveness. The second study estimated the price thresholds below which novel rifampicin-susceptible tuberculosis and rifampicin-resistant tuberculosis regimens would be cost-neutral or cost-effective.^11^

The target regimen profiles proposed here aim to guide the development of new tuberculosis treatment regimens, considering end-user needs and real-world conditions. We established a list of 13 characteristics – eight of which are common to all target regimen profiles (target population; populations of special interest; drug–drug interaction and metabolism; forgiveness of the regimen; number of component drugs; formulation or dosage form, dosing frequency and route of administration; propensity to develop resistance; and stability or shelf life); and five of which are regimen-specific (indication and need for drug susceptibility testing; efficacy; duration; safety, monitoring and tolerability; and pill burden)^11^ – and identified minimal and optimal targets for each. The expectation is that any regimen that is developed will meet most of the minimal requirements and as many of the optimal requirements as possible.

Based on this preliminary work, we developed an initial draft document that was shared with the group for revision and comments. We then divided into three subgroups who worked through virtual consultative meetings on each of the target regimen profiles, leading to a new version that was posted by WHO for public comment in February 2023. In March 2023, our group met to discuss the latest draft and reached full consensus on the contents of the document.

## Common regimen characteristics

### Target population

Regimens should be intended for all population groups, irrespective of age, site of disease, clinical severity and co-morbidities. The regimens must have an acceptable safety profile, be well tolerated and efficacious in all these groups (including neonates, infants, children, women of reproductive age, and pregnant and lactating women),^12^ as well as in those with comorbid conditions. Treatment developers should initiate paediatric studies as soon as a drug shows promising efficacy and safety in Phase 2 adult trials.^13^

### Populations of special interest

Human data indicate that the treatment does not cause any increased risk of structural abnormalities in the fetus, and the drugs are safe for women of childbearing potential, and pregnant and lactating women. The component drugs should be compatible with common forms of hormone-based birth control for women of reproductive age who do not wish to become pregnant.

### Drug–drug interaction and metabolism

New chemical entities should have minimal or no drug–drug interaction with the other components of the regimen combination. For people living with human immunodeficiency virus undergoing antiretroviral therapy, drug–drug interaction studies should be initiated as soon as doses are known; ideally, no dose adjustments as a result of drug–drug interactions should be required.^14^

### Forgiveness of the regimen

Imperfect adherence increases the risk of unfavourable treatment outcomes.^15^ On this basis, forgiveness of a regimen, defined as “the degree to which regimen efficacy is unaffected by suboptimal adherence,”^11^ was introduced as a new regimen characteristic. Because it can be challenging to ensure full daily drug administration under programmatic conditions, highly forgiving regimens are desirable.

### Number of component drugs

Regimens containing three to four drugs would provide the best balance to ensure high efficacy and minimize the risk of developing drug resistance. Further, limiting the number of component drugs in a regimen helps to minimize pill burden and safety risks, and facilitates drug coformulation.

### Formulation

Formulation should be all-oral, with simple, age- or weight-based dose adjustment. Fixed-dose combination formulations as well as child-friendly formulations are strongly encouraged.

### Propensity to develop resistance

Drugs included in the treatment regimen should protect each other against emergence of resistance; they should therefore have different targets,^16^ have different pharmacokinetic–pharmacodynamic properties, be active and synergistic at the lesion site level,^17^ and have half-lives that are well matched to reduce the risk of functional monotherapy.

### Stability and shelf life

Ideally, all component drugs should be stable for at least 5 years in climate zones III and IV (i.e. 30 °C and 75% relative humidity).

## Target regimen profiles^11^

### Rifampicin-susceptible tuberculosis

Despite its wide availability, low cost and high efficacy, the current 6-month treatment of rifampicin-susceptible tuberculosis has several limitations, including adverse events, drug–drug interactions and adherence requirements. A 4-month regimen was recommended by WHO as non-inferior to the 6-month regimen in 2020, but its uptake remains limited because of its high cost, lack of a fixed-dose combination and concerns about the use of a fluoroquinolone.^11^

#### Indication and drug susceptibility testing

The rifampicin-susceptible tuberculosis regimen is indicated for patients with active tuberculosis disease caused by rifampicin-susceptible *Mycobacterium tuberculosis* strains, including forms with monoresistance to isoniazid, pyrazinamide and ethambutol (the other drugs included in the current 6-month treatment regimen).

#### Efficacy and duration

The current 6-month regimen of isoniazid, rifampicin, pyrazinamide and ethambutol has 90–95% efficacy under trial conditions.^18^ The efficacy and duration targets aim to improve on the current 4-month WHO-recommended treatment (comprising the drugs isoniazid, rifapentine and moxifloxacin, with the addition of pyrazinamide in the first 2 months). However, the optimal target is based on the possible development of a 2-month regimen, as recently shown within the context of a sustained treatment monitoring strategy trial.^19^

#### Safety and tolerability

Although serious adverse events with the current standards of care are uncommon, important tolerability issues affect adherence and, as a result, effectiveness. The safety and tolerability of a new rifampicin-susceptible regimen should be at least equal to, and ideally better than, the current standards of care. Demands for active clinical and laboratory monitoring for drug toxicity should be minimized.

#### Pill burden

A regimen would ideally comprise just one pill per day; minimally, the pill burden should not be greater than for the 6-month regimen of isoniazid, rifampicin, pyrazinamide and ethambutol. 

### Rifampicin-resistant tuberculosis

In 2020, the global success rate for treatment of multidrug-resistant (MDR) tuberculosis (i.e. tuberculosis that is resistant to both rifampicin and isoniazid) was 60%.^20^ Treatment typically lasted 9–18 months and included a large number of drugs, resulting in high pill burden and frequent side-effects that often led to discontinuation of treatment. The recently recommended bedaquiline, pretomanid, linezolid and moxifloxacin regimen promises improvements; however, the safety profile is still suboptimal^21^ and careful implementation will be important to prevent emergence of resistance to component drugs.^22^

#### Indication and drug susceptibility testing

Under the minimal requirement, a rifampicin-resistant tuberculosis regimen is indicated for patients with active tuberculosis disease caused by rifampicin-resistant strains, with or without isoniazid resistance. Under the optimal requirement, the regimen is indicated for patients with active tuberculosis caused by either MDR tuberculosis, pre-extensively drug-resistant tuberculosis (caused by strains that are both MDR- and fluoroquinolone-resistant) or extensively drug-resistant tuberculosis (caused by strains resistant to any fluoroquinolones and at least one other Group A drug).^23^ Drug susceptibility testing is essential before initiation of treatment to establish the resistance pattern of the strains and guide the composition of the regimen. Under the optimal requirement, susceptibility to the drugs in the regimen should be established through appropriate phenotypic or genotypic testing. In all cases, usage should be consistent with principles of good antibiotic stewardship.^24^

#### Efficacy and duration

The 2022 WHO tuberculosis treatment guideline update recommends the 6-month bedaquiline, pretomanid, linezolid and moxifloxacin regimen for most adult patients with tuberculosis that is MDR or rifampicin resistant. This regimen is approximately 90% efficacious.^25^ Consequently, a new rifampicin-resistant-tuberculosis regimen should, at a minimum, have an efficacy at least as good as this newly recommended regimen and a similar duration; optimally, it should have a better efficacy and shorter duration than the 2022 regimen.

#### Safety and tolerability

The minimum and optimum targets are identical: it is suggested that the incidence and severity of adverse events with any new MDR-tuberculosis regimen should be lower than for the current regimens to guarantee best tolerability, acceptability and effectiveness under programmatic conditions. In the pivotal trial of the bedaquiline, pretomanid, linezolid and moxifloxacin regimen, adverse events of at least grade 3 occurring during treatment or up to 30 days after treatment were observed in 18% of patients (mainly hepatic, pancreatic or haematological disorders).^3^ The proportion of patients discontinuing this regimen because of non-tolerability was 5%.^3^

#### Pill burden

A regimen would ideally comprise not more than four to five pills per day; minimally, the pill burden should not be greater than five to seven pills per day. 

### Pan-tuberculosis treatment

#### Indication and drug susceptibility testing

A regimen containing fully novel compounds with no cross-resistance could be used as a first-line tuberculosis regimen in individuals with tuberculosis disease without prior knowledge of the patient’s drug resistance profile. This approach would allow treatment to begin without delay while drug susceptibility testing is sought. Such a pan-tuberculosis regimen would be particularly useful in areas with a high prevalence of drug resistance and low availability of, or low access to, rapid drug susceptibility testing; current practice in such areas means that patients may be treated with inappropriate regimens and may continue to transmit disease and generate additional drug resistance. Under these conditions, population-level drug resistance surveillance will be an important component of the pan-tuberculosis strategy.

#### Efficacy and duration

Considering that a pan-tuberculosis regimen would be used to treat both rifampicin-susceptible and rifampicin-resistant tuberculosis, efficacy should be at least as good as the current rifampicin-susceptible tuberculosis regimen, with a duration of no more than 3–4 months. Optimally, efficacy should be better than the current regimen and, based on recent results indicating that a carefully applied monitoring strategy can make it possible to provide a cure with a 2-month regimen,^19^ duration should be equal to, or less than, 2 months.

#### Safety and tolerability

In line with the recommendations for the rifampicin-susceptible and rifampicin-resistant tuberculosis regimens, and considering that the pan-tuberculosis regimen will be provided to a larger proportion of patients with rifampicin-susceptible than rifampicin-resistant tuberculosis, it is expected that the incidence and severity of adverse events should be lower and tolerability better than for the rifampicin-susceptible tuberculosis regimen, as both a minimal and an optimal target.

#### Pill burden

A regimen would ideally comprise just one pill per day; minimally, the pill burden should not be greater than for the 6-month regimen of isoniazid, rifampicin, pyrazinamide and ethambutol. 

## Cross-cutting aspects

We discuss several key aspects that are relevant across all three regimens: drug susceptibility testing; adherence and forgiveness; treatment strategies; post-tuberculosis lung disease; and cost and access considerations.

### Drug susceptibility testing

Phenotypic or genotypic tests should be available at time of regimen introduction for both individual patient care and population-level surveillance. Drug developers need to support this by establishing minimum inhibitory concentration distributions and drug susceptibility testing interpretative criteria. To allow an understanding of resistance mechanisms, the identification of target genes and the development of rapid drug susceptibility testing, drug developers must also provide access to resistant mutants and drug compounds with detailed accompanying information (including stability, storage and solubility).

### Adherence and forgiveness

Poor adherence affects treatment outcomes and increases the risk of developing resistance, although uncertainty remains with regards to the magnitude of this effect.^26^ A meta-analysis of trial participants receiving isoniazid, rifampicin, pyrazinamide and ethambutol suggested that those who missed 10% or more of treatment doses had a 5.9-fold greater risk of unfavourable treatment outcome.^15^ This outcome suggests that imperfect adherence has a major impact on treatment outcomes and that, with the current dosing, such a regimen is unforgiving. However, studies to date present conflicting findings, showing considerable uncertainty about the degree to which improving adherence may enhance treatment outcomes.^27^ High levels of poor adherence have been observed outside of well-controlled clinical trials;^27^ developing more forgiving regimens that retain high proportions of patients cured under such circumstances – and are therefore effective and not just efficacious – is an important priority.

### Treatment strategies

Beyond the specific characteristics of treatment regimens, novel treatment strategies such as stratified medicine approaches may be implemented to maximize the benefits and minimize the harms of treatment. Re-analyses of clinical trial data suggest that a difficult-to-treat subset of patients with rifampicin-susceptible tuberculosis may require longer than 6 months of the regimen to reach target cure, while most patients could be successfully treated in 4 months or less.^15^^,^^28^ Further research is needed to investigate the use and applicability of stratified approaches for tuberculosis treatment under programmatic conditions.

### Post-tuberculosis lung disease

There is increasing recognition that pulmonary tuberculosis (both rifampicin-susceptible and rifampicin-resistant) may result in clinically significant lung injury and functional impairment, termed post-tuberculosis lung disease, even in patients whose treatment is otherwise successful. Developers are encouraged to incorporate evaluation of lung injury and functional impairment into trial designs. As our understanding of post-tuberculosis lung disease grows, developers may anticipate the inclusion of a new characteristic in future target regimen profiles: the ability to limit lung injury and functional impairment, and therefore the emergence of post-tuberculosis lung disease.

### Cost and access considerations

Given the significant role of public financing for tuberculosis research and development, new products should be appropriately priced to reflect overall investments by global public and public–private actors, including governments, philanthropists, and other research and product sponsors. Any resulting product should deliver a public return on investment, and be linked to public health-driven priorities and application of the core principles of affordability, effectiveness, efficiency and equity. Developers should aim for new tuberculosis regimens that are cost-neutral, if not cost-saving, to health systems, considering both drug and non-drug costs. Our modelling analysis suggests that costlier regimens can still be cost-neutral if optimal regimen targets are met, as this would reduce non-drug costs (e.g. treatment monitoring visits or patient adherence support).^11^ There should be collective efforts to ensure accelerated development, commercialization and scale-up of affordable regimens satisfying the criteria laid out in these target regimen profiles.

## Discussion

The 2023 update of the WHO target regimen profiles for tuberculosis treatment highlights key priorities for the next generation of tuberculosis treatments. However, it might not be feasible to fully meet all requirements simultaneously; developers may have to prioritize one or several characteristics over others, taking into consideration the intrinsic properties of the drugs included in the regimen and the health needs being targeted. Several perspectives can guide developers handling these trade-offs, including the clinical perspective (where probability of cure is a priority); the economic perspective (relating to the costs to health systems and patients); the patients’ perspective (drawing on experiences of tuberculosis survivors); and the expected long-term impact at the population level.

We undertook a modelling analysis to estimate the impact on health outcomes and costs of improving various regimen characteristics.^11^ In this analysis, ease of adherence (defined as the proportion of prescribed doses that patients take effectively while still on treatment) had the greatest influence on proportion of patients cured. For regimens meeting all the minimal characteristics of their respective target regimen profiles, complete adherence (as might be achieved via a long-acting injectable) increased the percentage of patients cured from 83% to 93% for rifampicin-susceptible tuberculosis, and from 74% to 85% for rifampicin-resistant tuberculosis.^11^ Improving the efficacy of the regimen was the next most influential characteristic, especially for rifampicin-resistant tuberculosis. Of note, shortening treatment duration had a smaller effect on cure than other modelled characteristics, but had the greatest influence on reducing costs.^11^ This finding aligns with observations from the stakeholders’ survey, in which respondents expressed that regimens should primarily demonstrate strong efficacy (even at the expense of longer duration), followed by safety, treatment duration, frequency of intake and pill burden.

Drug developers should therefore consider the capacity of their proposed regimen under development to be best used in programmatic conditions. Indeed, beyond the intrinsic pharmacological and antibacterial characteristics of new regimens, their clinical and public health impact will also depend on operational and epidemiological factors that affect regimen use (e.g. the ability to access, afford and distribute new regimens; the availability of clear guidance for clinicians; the background prevalence of antimicrobial resistance; and the development of resistance to new drugs).^29^ For these reasons, the respective characteristics to be considered at the developmental stage should not be dissociated from the factors to be considered at implementation stage; we need effective regimens that are accessible, affordable and work for people and programmes, not just efficacious regimens that work well in clinical trials. To simplify regimen development, and ensure that products are fit for purpose and can meet the needs of affected communities (particularly in low-resource areas), developers should therefore (i) work within open collaborative models for tuberculosis research and development to enable early sharing of research knowledge; and (ii) consult affected communities to ensure that needs and priorities of patients are driving the final product and use-case.

This target regimen profile update also highlights the deleterious impact of drug resistance. Developers should work in partnership with reference laboratories and diagnostic manufacturers to identify protocols and standards for phenotypic and genotypic tests, providing the necessary tools (compounds, mutants if available, information on drug resistance mechanisms) in parallel with drug development. A pan-tuberculosis regimen could facilitate implementation of tuberculosis treatment, but should be considered as a better first-line regimen for all and not a drug-susceptibility-testing-free regimen. The 2016 target regimen profile encouraged a shift in focus from drug to regimen development; the 2023 profiles draw awareness to considering not just regimens but treatment strategies to optimize care of patients.

In conclusion, the revised target regimen profiles described here represent an important step towards the development of new regimens for the treatment of all forms of tuberculosis. These profiles are expected to serve as a reference for research consortia involving drug developers, academics, public health institutions, nongovernmental organizations and civil society organization representatives, so that better treatment of all forms of tuberculosis is available to achieve the targets of the WHO end tuberculosis strategy.^30^
